# Younger age of onset in familial amyotrophic lateral sclerosis is a result of pathogenic gene variants, rather than ascertainment bias

**DOI:** 10.1136/jnnp-2018-319089

**Published:** 2018-09-30

**Authors:** Puja R Mehta, Ashley R Jones, Sarah Opie-Martin, Aleksey Shatunov, Alfredo Iacoangeli, Ahmad Al Khleifat, Bradley N Smith, Simon Topp, Karen E Morrison, Pamela J Shaw, Christopher E Shaw, Sarah Morgan, Alan Pittman, Ammar Al-Chalabi

**Affiliations:** 1 Department of Basic and Clinical Neuroscience, Maurice Wohl Clinical Neuroscience Institute, King’s College London, London, UK; 2 Department of Neurology, King's College Hospital, Denmark Hill, London, UK; 3 Department of Biostatistics and Health Informatics, Institute of Psychiatry, Psychology and Neuroscience, King’s College London, London, UK; 4 Faculty of Medicine, University of Southampton, University Hospital Southampton NHS Foundation Trust, Southampton, UK; 5 Sheffield Institute for Translational Neuroscience (SITraN), University of Sheffield, Sheffield, UK; 6 Institute of Psychiatry, Psychology and Neuroscience, UK Dementia Research Institute, Maurice Wohl Clinical Neuroscience Institute, King’s College London, London, UK; 7 Department of Molecular Neuroscience, Institute of Neurology, UCL, Queen Square, London, UK

## Abstract

**Objective:**

Amyotrophic lateral sclerosis (ALS) is a rapidly progressive neurodegenerative disease of motor neurons with a median survival of 2 years. Familial ALS has a younger age of onset than apparently sporadic ALS. We sought to determine whether this younger age of onset is a result of ascertainment bias or has a genetic basis.

**Methods:**

Samples from people with ALS were sequenced for 13 ALS genes. To determine the effect of genetic variation, age of onset was compared in people with sporadic ALS carrying a pathogenic gene variant and those who do not; to determine the effect of family history, we compared those with genetic sporadic ALS and familial ALS.

**Results:**

There were 941 people with a diagnosis of ALS, 100 with familial ALS. Of 841 with apparently sporadic ALS, 95 carried a pathogenic gene variant. The mean age of onset in familial ALS was 5.3 years younger than for apparently sporadic ALS (p=6.0×10^−5^, 95% CI 2.8 to 7.8 years). The mean age of onset of genetic sporadic ALS was 2.9 years younger than non-genetic sporadic ALS (p=0.011, 95% CI 0.7 to 5.2 years). There was no difference between the mean age of onset in genetic sporadic ALS and familial ALS (p=0.097).

**Conclusions:**

People with familial ALS have an age of onset about 5 years younger than those with apparently sporadic ALS, and we have shown that this is a result of Mendelian gene variants lowering the age of onset, rather than ascertainment bias.

## Introduction

Amyotrophic lateral sclerosis (ALS), also known as motor neuron disease, is a rapidly progressive and paralysing illness resulting from degeneration of upper and lower motor neurons; it has no cure and the median survival is 2 years.^1^ The mean age of onset in clinic populations is about 58 years,^2^ and in population studies about 64,^3^ but it can affect people of any age.

The age of onset of people with familial ALS is widely reported as being about 10 years younger than for those with apparently sporadic ALS.^4–6^ This may be a result of the greater awareness of those who have already experienced ALS in their family, leading them to seek medical attention sooner, and the greater likelihood of recognition of the diagnosis by medical professionals when there is an existing family history. Such ascertainment bias has been proposed as the mechanism for the genetic phenomenon of anticipation, in which genetic diseases seem to occur at younger ages in succeeding generations.^7^ Another explanation, however, is that large-effect Mendelian ALS genes themselves lower the disease age of onset. Studies aiming to investigate genetic factors that influence ALS phenotypes, including age of onset, have yet to yield replicable positive results.^8^

Familial ALS has been described since the mid-1800s, and the rate is reported as 5%,^9^ 10%^10^ or as high as 20%.^11^ The reasons for the discrepancy probably relate, at least in part, to differing personal definitions of a positive family history.^12^ People with ALS but without a family history are described as having sporadic or isolated ALS. Sporadic ALS is sometimes conflated with non-genetic ALS, but familial ALS may be missed, for example, because of the uncertainty around what should constitute a relevant family history, and apparently sporadic ALS may sometimes result from large-effect, rare, Mendelian gene variants.^13–15^ The result is that there are three genetic groups of people with ALS: those with a family history of disease (group 1), those with no family history but a known genetic cause (group 2), and those with no family history and no known genetic cause (group 3). The relative age of onset would be expected to differ between these groups depending on whether ascertainment bias or genetic effects are driving the earlier onset. If ascertainment bias is the explanation for a younger age of onset in familial ALS, group 1 should have a younger age of onset than group 2, because the only difference is knowledge of family history (both share a genetic basis), whereas if genetic causes are the explanation, group 2 should have a younger age of onset than group 3, because the only difference is a genetic basis of the disease (in both there is no known family history).

We therefore sought to determine whether the younger age of onset observed in those with familial ALS is a result of ascertainment bias.

## Methods

### Data sources

Patients of European ancestry who had contributed to the UK National DNA Bank for Motor Neuron Disease Research were analysed. Age of onset and family history were obtained for each person.

### Determination of pathogenic ALS gene variants

A panel of 13 ALS genes was tested (*ANG*, *C9orf72*, *DAO*, *DCTN1*, *FUS*, *OPTN*, *PFN1*, *SOD1*, *SQSTM1*, *TARDBP*, *UBQLN2*, *VAPB* and *VCP*), selected for harbouring large-effect, rare, Mendelian ALS gene variants.^16 17^ Pathogenicity was defined based on previously reported association with ALS, or detection in cases, but not in controls or several control databases.^17^ Because of the rapid rate of advance of ALS genetics, more recently identified genes, such as *TBK1*, were not studied.^18^ DNA was sequenced using Illumina MiSeq technology by means of the Illumina TruSeq Custom Amplicon Assay, as previously described,^17^ and repeat primed PCR used to assay the hexanucleotide repeat expansion in the *C9orf72* gene.^19^

Patients were classified into three groups: group 1, familial ALS; group 2, apparently sporadic ALS with a genetic basis; group 3, apparently sporadic ALS without an identified genetic basis.

### Statistical analysis

The mean age of onset between groups was compared with a *t*-test. Uncorrected p values are reported. To estimate the size of any ascertainment bias observed, the median time between symptom onset and diagnosis was compared between those with familial ALS and those with apparently sporadic ALS, in a Mann-Whitney U test. Analyses were performed in SPSS V.24.0.

## Results

There were 941 people with ALS included in the study, 841 with apparently sporadic ALS and 100 with familial ALS (table 1). There were 95 people with apparently sporadic ALS with a presumed pathogenic ALS gene variant (table 2) and 746 without.

**Table 1:** Mutations identified in those with familial amyotrophic lateral sclerosis (ALS)

Gene	Variant	Cases (n)
*C9orf72*	Expansion mutation	34
*DAO*	p.R199W	1
*FUS*	p.R521H	2
	p.R514G	1
*SOD1*	p.C112Y	1
	p.D91A	1
	p.L107F	1
	p.D102N	1
	p.D102G	1
	p.I114T	2
	p.D77Y	2
*SQSTM1*	p.K238E	2
*TARDBP*	p.N378D	2
	p.A90V	1
	p.M337V	1
	p.G348V	1
	p.Y374X	1
*UBQLN2*	p.P497H	1
No mutation identified		44
Total		100

95 of the 100 familial ALS cases were tested for the *C9orf72* expansion mutation.

**Table 2:** Mutations identified in those with apparently sporadic amyotrophic lateral sclerosis

Gene	Variant	Cases (n)
*ANG*	p.K78E*	2
	p.R146H	1
*C9orf72*	Expansion mutation*	36
*DAO*	p.R199Q	1
*DCTN1*	p.V1081M	1
	p.T12A	1
	p.R785W	1
*FUS*	p.R269W	1
	p.R521C	1
	p.P431L	1
	p.R521L	1
	p.G507D	1
	p.221_221del	1
*OPTN*	p.R545Q	1
	Exon 7/Intron 7 splice site	1
	p.Q441X	1
	p.E380fs	1
	p.V295F	1
	p.N303K	1
	p.G509R	1
	p.A184V*	1
	p.K413fs*	1
	p.R271H*	1
	p.F226S	1
*PFN1*	p.E117D*	1
	p.E117G*	1
*SOD1*	p.D91A	1
	p.T40A*	1
	p.S135G*	1
	p.I114T	2
	p.L9V	1
*SQSTM1*	p.K238E*	7
	p.P392L*	2
*TARDBP*	p.G287S*	3
	p.A90V	1
*UBQLN2*	p.A603D	1
	p.T334M	3
	p.L87F	1
	p.P497H	1
	p.496_499del	1
*VAPB*	p.158_159del*	7
	p.M170I*	5
	p.R184Q	1
*VCP*	p.I114V	1
	p.G523V	1
Total		103 variants (95 people)

In some cases, an individual had more than one pathogenic gene variant; such cases are indicated by an asterisk and counted for each variant. Three of these had *C9orf72* expansion as one of the variants.

The mean age of onset for those with familial ALS was 56.2 years (SD 12.1), all apparently sporadic ALS 61.5 years (SD 11.0), genetically determined apparently sporadic ALS 58.9 years (SD 10.3) and apparently sporadic ALS without a genetic basis 61.8 years (SD 11.1).

The mean age of onset in familial ALS was 5.3 years younger than for apparently sporadic ALS (p=6.0×10^−5^, 95% CI for the difference 2.8 to 7.8 years), confirming previous observations that familial ALS has a younger age of onset.

There was no difference between the mean age of onset of people with familial ALS (group 1) and those with genetic apparently sporadic ALS (group 2; p=0.097), suggesting that ascertainment bias plays little role in the observed younger age of onset. The mean age of onset of those with genetic apparently sporadic ALS (group 2) was 2.9 years younger than those with non-genetic sporadic ALS (group 3; p=0.011, 95% CI for the difference 0.7 to 5.2 years), consistent with the hypothesis that genetic burden lowers the age of onset. Combining the genetic groups and comparing them with the non-genetic ALS group confirmed the finding, with a mean age of onset of 3.4 years younger for the genetic groups than the non-genetic group (p=2.0×10^−6^, 95% CI for the difference 2.5 to 6.1).

The median time between symptom onset and diagnosis was 3 months shorter for familial ALS than apparently sporadic ALS (p=0.012).

## Discussion

We have confirmed that people with familial ALS have an age of onset about 5 years younger than those with apparently sporadic ALS, as opposed to the widely quoted 10 years, and we propose that this up-to-date figure obtained from a large data set should be used in the clinical setting, particularly when communicating with patients. We have shown that this younger age of onset in familial ALS is a result of Mendelian gene variants lowering the actual age of onset, rather than ascertainment bias resulting in quicker recognition of symptoms. Ascertainment bias does exist, in that people with a family history of ALS are diagnosed on average 3 months more quickly than those without, but the ascertainment bias has no effect on age of onset.

Our findings are consistent with recent findings in which the multistep model of ALS^20^ was applied to three known genetic causes of ALS: mutation in *SOD1*, mutation in *TARDBP* (coding for TDP43) and pathological expansion of a hexanucleotide repeat in the *C9orf72* gene.^21^ These gene variants account for more than one step in the pathological process leading to ALS, leaving fewer risk factors to encounter before onset and therefore making a younger age of onset more likely. One might also expect some genes or gene variants to show a characteristic spectrum of age of onset, which will become apparent as our genetic knowledge of ALS increases. A similar situation may also exist in other neurodegenerative diseases in which those with a family history have a younger onset than those without, such as Alzheimer’s disease and Parkinson’s disease.

A weakness of this study is that we only ascertained a genetic basis for ALS using a panel of 13 genes. Even with this restricted set, however, we were able to show that the reduced age of onset is a result of Mendelian disease genes. Furthermore, it is only now, with the availability of high-throughput gene sequencing, that a study like this is possible, since the prospect of sequencing a panel of 13 genes in more than 900 people would not have been feasible a few years ago.

Genetic modulators of the age of ALS onset have been studied before,^8 22–27^ but not replicated. The explanation for why these have not been replicated may be that genetic risk factors themselves lower the age of onset and other genetic variants that do not influence risk have only a limited effect on age of onset, making them difficult to detect, even with large sample sizes.

Although we have identified this genetic effect on age of onset using large-effect, rare variants, the liability threshold model of disease^28 29^ would suggest that all genetic contributors to ALS risk lower age of onset, even common variants that have a small effect on risk. In the liability threshold model, there is a normal distribution of the burden of genetic and environmental risk factors (liabilities) across the population. Most individuals who are unaffected will possess some degree of liability; however, only individuals with a burden of risk factors sufficient to cross a threshold develop a disease. A consequence of the model is that affected individuals with the least at-risk phenotype are also those with a higher liability than average, since the threshold must be higher for a low-risk phenotype (that is why it is low risk), and yet they still have sufficient burden to cross it. One of the strongest risk factors for ALS is increased age, and therefore younger patients must carry a higher burden of risk factors. Thus, genetic burden is likely to be higher in younger patients, and conversely the age of onset will be lower in those with a higher genetic burden. One study has shown that people with ALS harbouring variants in more than one gene had a disease onset 10 years earlier than those with variants identified in only one gene, supporting the idea that genetic burden influences age of onset.^30^

Our findings show that Mendelian gene variants are associated with a younger age of onset in ALS, regardless of family history. Further studies are needed to elucidate the mechanisms by which this occurs.
